# Supplementary material for: MetaRibo-Seq measures translation in microbiomes
Source: Nat Commun. 2020 Jun 29;11:3268. doi: 10.1038/s41467-020-17081-z (PMC7324362; doi:10.1038/s41467-020-17081-z)
Supplement: Supplementary file 10 — Supplementary Data 7 [file 41467_2020_17081_MOESM10_ESM.zip › File2/Confidence_High_Taxonomy/111919_out.krona.html]

Javascript must be enabled to view this page.

members
magnitude
magnitudeUnassigned
count
unassigned
taxon
rank

111919\_out

3

3
superkingdom
2

phylum
3
1239

526524
3
class

order
3
526525

128827
family
3

1573535
genus
3

1735

SRS015431\_contig\_number\_86579SRS019068\_contig\_number\_18440SRS1041037\_contig\_number\_8017
3
species
